# A novel *BCR-ABL1* fusion gene with genetic heterogeneity indicates a good prognosis in a chronic myeloid leukemia case

**DOI:** 10.1186/s13039-017-0322-8

**Published:** 2017-05-19

**Authors:** Fen Zhou, Runming Jin, Yu Hu, Heng Mei

**Affiliations:** 1 Institute of Hematology, Union Hospital, Tongji Medical College, Huazhong University of Science & Technology, 1277 Jiefang Avenue, Wuhan, Hubei 430022 China; 2 Department of Pediatrics, Union Hospital, Tongji Medical College, Huazhong University of Science and Technology, 1277 Jiefang Avenue, Wuhan, Hubei 430022 China

**Keywords:** CML, *BCR-ABL1*, NGS, SH3 domain, Genetic heterogeneity

## Abstract

**Background:**

Chronic myelogenous leukemia (CML) is a pluripotent hematopoietic stem cell disorder caused by the fusion of the *BCR* and *ABL1* genes. Quantitative RT-PCR (qRT-PCR) is a routinely performed screening technique to identify *BCR-ABL1* fusion genes, but a limitation of this method is its inability to recognize novel fusions that have not been previously characterized. Next-generation sequencing (NGS) is an effective and sensitive detection method for the determination of novel *BCR-ABL1* fusion genes as well as previously characterized ones. The oncoprotein tyrosine kinase BCR-ABL1 is a constitutively active kinase involved in the activation of a number of signaling pathways, and it has been the therapeutic target for tyrosine kinase inhibitors (TKIs) such as imatinib. Reports have presented opposing viewpoints about the effect of the disrupted Src homology 3 (SH3) domain on TKI efficacy.

**Findings:**

We here report that using NGS we identified a novel *BCR-ABL1* fusion gene with breakpoints in the *BCR* intron 14 and the *ABL1* intron 2, leading to partial deletion of its SH3 domain. In the present case, the patient received targeted therapy with the TKI imatinib at 400 mg/day and no adverse reaction was reported. The patient eventually entered remission with decreased proliferation of karyocytes and granulocytes. We also identified mutations in genes, including *TP53*, *FLT3*, *ASXL1*, *SETBP1*, *CEBPA* and *CBL,* that seemed to have an influence on the outcome of TKI therapy targeting the BCR-ABL1 protein.

**Conclusions:**

Together with previously reported results, it is clear that the genetic heterogeneity of CML patients significantly affects the presentation of the disease and its progression and therefore should inform the design of the therapeutic strategy.

## Background

CML, a clonal hematopoietic stem cell disorder, is characterized by the fusion of the Abelson gene (*ABL1*) on chromosome 9q34 with the breakpoint cluster region (*BCR*) gene on chromosome 22q11.2, which is known as the Philadelphia translocation [1]. This molecular rearrangement results in formation of the *BCR-ABL1* oncogene. Its translation product, oncoprotein BCR-ABL1, shows enhanced tyrosine kinase activity and plays a critical role in the transformation of hematopoietic stem cells through activation of a number of signaling pathways [2, 3]. According to the locations of the breakpoints in the *BCR* and *ABL1* genes, fusion genes are divided into many known species, such as e13-a2, e14-a2, e19-a2 and e1-a2 [4, 5], as well as other uncharacterized fusion genes. Routine screening procedures, such as multiplex qRT-PCR assays, are designed to detect previously characterized *BCR-ABL1* fusion transcripts and, thus, have limited ability to detect novel ones. That problem can be resolved with the application of NGS technology since it can identify these novel mutations undetectable by routine screening procedures [6–8], as well as those previously characterized. Thus NGS plays an important role in genetic diagnostics and is helpful for a better understanding of the cancer genome.

An article [9] entitled “A novel *BCR-ABL1* fusion gene identified by next-generation sequencing in chronic myeloid leukemia” has been published recently. Here, we report that we have also identified this novel *BCR-ABL1* fusion gene in another patient using NGS technology. We also report that this patient carries a different set of genetic mutations than those that impacted the outcome of TKI imatinib treatment in the Lyu et al. report [9]. Comparison of these studies demonstrates that genetic heterogeneity can be a key influencing factor in the therapeutic resolution of CML.

## Results

**Fig. 1:**
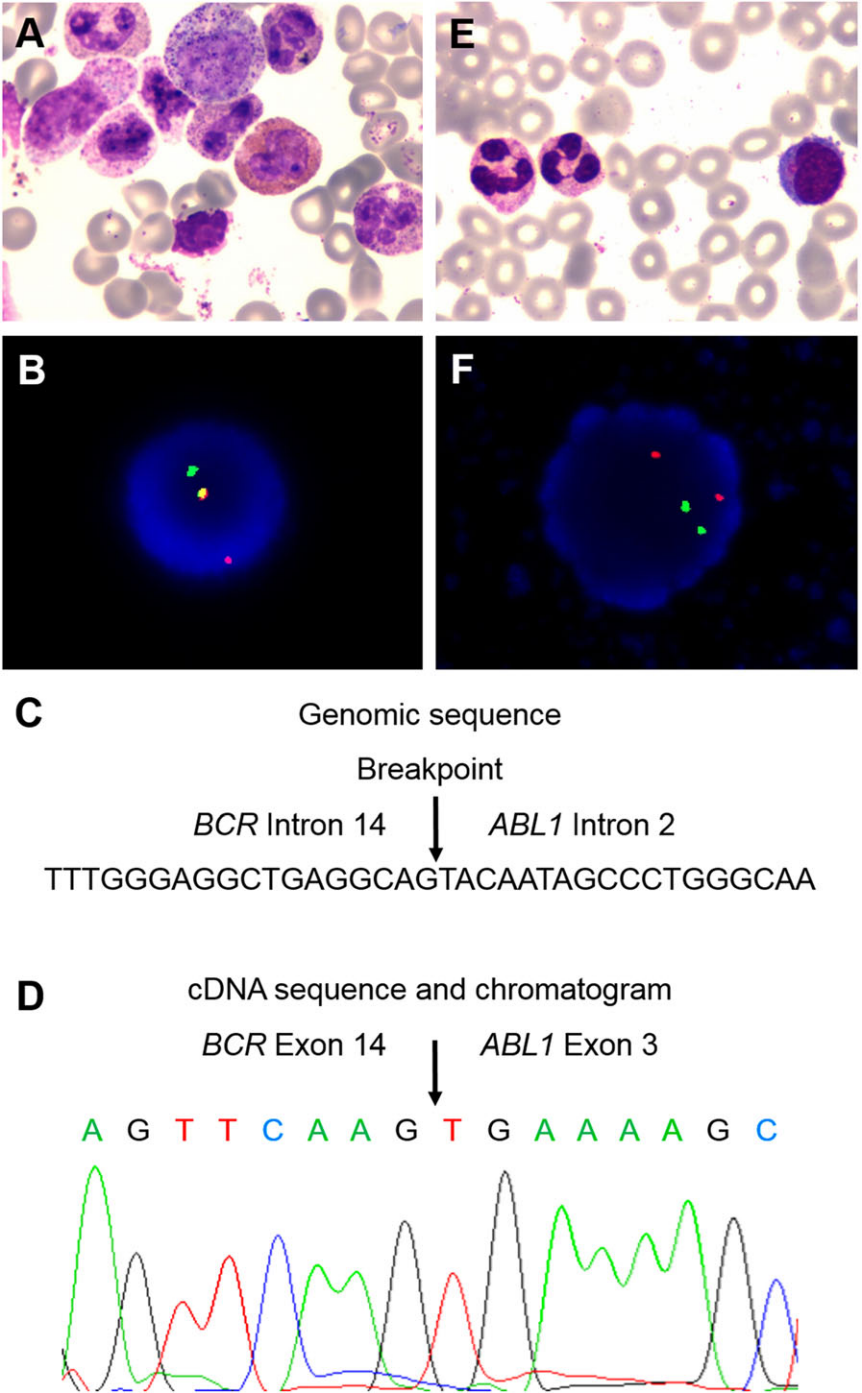
Summary of FISH and molecular studies. **a** Image of bone marrow aspiration (400x) showing hypercellularity with elevated level of myeloblasts, eosinophils, and basophils. **b** FISH analysis. Separated *green* and *red* signals indicate probe-targeted sequences located on different chromosomes in a normal nucleus. *Yellowish* signal formed from the colocalization of *green* and *red* fluorescent signals indicates the fusion of *BCR* and *ABL1* genes. **c** The breaking point (or fusion junction) and flanking sequences from *BCR* Intron 14 and *ABL1* intron 2. **d** *BCR-ABL1* cDNA sequence around the fusion junction and related chromatogram are shown. The junctions are indicated with *arrows*. **e** Image of bone marrow aspiration after imatinib targeted therapy. **f** FISH analysis after imatinib targeted therapy

Our patient is a 62-year-old male who presented at our hospital in February 2016 with intermittent nasal bleeding that had exceed 1 month in duration. After hospitalization, we determined the patient had a significantly elevated platelet level that increased the risk of bleeding and thrombosis to a life-threatening level. No superficial lymph nodes were detected anywhere in the body. The patient was diagnosed with CML through blood and bone marrow examinations. Peripheral blood smear analysis indicated elevated levels of total white blood cells (WBCs, 55.24 g/L), neutrophils (34.58 g/L), thrombocytes (2597 g/L), and a normal level of hemoglobin (103 g/L). Bone marrow aspiration analysis revealed the active proliferation of bone marrow nucleated cells (BMNCs) and elevated proportions of eosinophils and basophils. Granulocytes accounted for 88% of WBCs due to the excessive proliferation of band granulocytes and segmented granulocytes (Table 1 and Fig. 1a). We also observed a decreased level of lymphocytes with normal morphology.

**Table 1:** Comparison of bone marrow aspiration analyses before and after therapy

Cell type	Cell count (%)		
	Reference (Mean ± sem)	Before therapy	After therapy
Myeloblast	0.64 ± 0.33	1.5	0
Promyelocyte	1.57 ± 0.60	1.0	0
Neutrophil			
N.myelocyte	6.49 ± 2.04	7.0	1
N.metamyelocyte	7.90 ± 1.97	6.5	1
N. band	23.72 ± 3.50	33.0	20
Neutrophil	9.44 ± 2.92	28.0	45
Eosinophil	0.86 ± 0.61	10.5	7
Basophil	0.03 ± 0.05	3.5	0
Lymphocyte	22.78 ± 7.04	4.0	20
Monocyte	3.00 ± 0.88	0	3

Bone marrow karyotype analysis showed a phenotype of 46,XY,t(9;22)(q34;q11.2) (Data not shown). Fluorescence in situ hybridization (FISH) analysis was then used to detect the fusion between *BCR* and *ABL1* genes, which were demonstrated as dots of yellowish fluorescent signals formed from the colocalization of the green (*BCR*) and red (*ABL1*) fluorescent signals. We found at least one yellowish fluorescent dot per cell in 44% of cells, representing the tumor cells with *BCR-ABL1* fusions (Fig. 1b). However, fluorescent qRT-PCR failed to detect the previously characterized *BCR-ABL1* fusion transcripts p190 (e1-a2), p210 (e13-a2 and e14-a2) and p230 (e19-a2). To further clarify the existence of a *BCR-ABL1* fusion in this patient, we conducted whole genome sequencing (WGS) analysis on a NGS platform. We detected a *BCR-ABL1* fusion gene with novel breakpoints in *BCR* intron 14 and *ABL1* intron 2 (Fig. 1c), confirming the fusion of *BCR* exon 14 (e14) and *ABL1* exon 3 (a3). The corresponding *BCR-ABL1* hybrid mRNA was eventually identified by RT-PCR with a pair of custom primers targeting e14 and a3, followed by Sanger sequencing (Fig. 1d). After 7 days of imatinib treatment, the disease was under control with an improved platelet count and the patient discharged. As an outpatient, he then continued treatment with imatinib at 400 mg/day, accompanied by sodium bicarbonate tablets at 3.0 g/day and allopurinol tablets at 0.3 g/day, with regular follow-up visits. After 4 months, we measured a significant decrease in bone marrow karyocyte proliferation with the reduced number of granulocytes now accounting for 67% of WBCs in this CML patient (Table 1, Fig. 1e). We saw a great improvement in disease progression – the patient achieved both hematologic and molecular remission (Fig. 1e–f).

## Discussion

Philadelphia translocation, formed by the junction of *BCR* and *ABL1* genes, has been proven to be involved in the carcinogenesis of CML. In this work, we have identified a novel *BCR-ABL1* fusion gene by NGS, together with other co-existing mutations, indicating that genetic heterogeneity is associated with the response to imatinib treatment for this CML case and may require optimization of the personalized therapeutic schedule targeting CML.

The introduction of small molecule TKIs has contributed to marked improvements in the therapeutic outcomes of CML by forcefully blocking phosphorylation by the BCR-ABL1 oncoprotein and inhibiting its cell signal transduction activity [10–12]. Imatinib is a tyrosine-kinase inhibitor used in the treatment of multiple cancers and was the first TKI to receive approval by the Food and Drug Administration for the treatment of patients with Philadelphia chromosome-positive (Ph+) CML [13, 14]. After detecting the novel *BCR-ABL1* fusion gene in our patient, imatinib was used at 400 mg/day as a targeted treatment. It has been reported that the SH3-SH2 (Src homology 3-Src homology 2) domain in the ABL protein plays a crucial role in regulating its tyrosine kinase activity [15]. The function of partial deletions of the SH3 domain, which is encoded by *ABL1* exons 2 and 3, remains controversial. The report from Lyu et al. showed that their patient was intolerant to a normal dosage of imatinib, indicating an interaction between this unusual therapy outcome and the incomplete SH3 domain caused by the deletion of *ABL1* exon 2 [9]. Our case differed from that of Lyu et al. in that our patient with the e14-a3 *BCR-ABL1* fusion gene was not refractory to or intolerant of imatinib treatment. The patient achieved both hematologic and molecular remission after 4 months of imatinib treatment. A previous study [16] reported that the STAT5 signaling pathway induced by the ABL1 SH3 domain plays a critical role in the anti-apoptotic activity and cell cycle progression involved in *BCR-ABL1* leukemogenesis. Thus the truncation of the SH3 domain caused by deletion of *ABL* exon 2 may result in the reduction of leukemogenesis. CML patients with an e13a3 fusion gene were found to have a good response to imatinib, and could achieve complete hematologic and cytogenetic remission [17].

Similar to previous results, we also detected nonsynonymous mutations in leukemic genes such as *TP53* (c.C215G: p.P72R) and *FLT3* (c.C680T: p.T227M) through NGS. Furthermore, we also found mutations in *ASXL1* (c.T2444C:p.L815P), *SETBP1* (c.G664A: p.A222T) (c.G3301A: p.V1101I) (c.C3388A: p.P1130T), *CEBPA* (c.570_571insCACCCG: p.H191delinsHPH) and *CBL* (c.C1858T:p.L620F) that co-existed with the *BCR-ABL1* fusion in our patient. *ASXL1* mutations are common in myeloid neoplasms, including myelodysplastic syndrome (MDS) [18, 19], chronic myelomonocytic leukemia (CMML) [20, 21], primary myelofibrosis [18, 22], and acute myeloid leukemia (AML) [19, 23]. *SETBP1* mutations have been identified in atypical chronic myeloid leukemia (aCML), which is a rare disorder of hematopoietic stem cells and shares clinical and laboratory features with CML but lacks the *BCR-ABL* fusion gene [24]. Other strongly linked hematological malignancies, such as chronic neutrophilic leukemia (CNL), CMML, unclassified MDS, myeloproliferative neoplasms (MPNs), and secondary acute myelocytic leukemia (AML) evolving from MDS [25–29], are also related to *SETBP1*. Despite the fact that mutations in both *ASXL1* and *SETBP1* are generally associated with an adverse prognosis [20, 21, 26, 30], our patient’s symptoms seemed not to be related to his mutations in these genes. CCAAT enhancer binding protein α (C/EBPα), a general inhibitor of cell proliferation and a tumor suppressor [31] plays a pivotal role in early granulocyte development. C/EBPα is one of the crucial transcription factors for myeloid cell development and has been found to be involved in hematopoietic differentiation. The mutation of its coding gene *CEBPA* results in dysregulation of transcription, translation or post-translational modifications. These disruptions cause disorders of differentiation and over proliferation of immature hematopoietic cells [32, 33]. In our patient, the outcome of imatinib therapy for CML suggests that his disease was not significantly affected by what we can consider to be ancillary mutations. Considering the results reported by Lyu et al. [9], it can be concluded that the variety of genetic mutations among individual CML patients may lead to different treatment outcomes of TKI therapies targeted for *BCR-ABL1*. More research is needed to illuminate the interactions between these uncommon mutations and the variety of *BCR-ABL1* fusion genes in CML.

## Conclusions

We report this case to demonstrate that by NGS we have detected the same *BCR-ABL1* fusion that disrupts the SH3 domain, as Lyu et al. [9]. Meanwhile, we also found numerous other mutations in genes such as *TP53*, *FLT3*, *ASXL1*, *SETBP1*, *CEBPA* and *CBL*, suggesting that CML may be more highly heterogeneous than previously appreciated. Our findings show that such genetic heterogeneity may significantly affect treatment outcomes and should therefore inform the therapeutic strategy. Since these conclusions remain speculative, more studies should be performed to characterize the various interactions between *BCR-ABL1* gene rearrangements and mutations in other oncogenes.

## Methods

### Detection of *BCR-ABL* fusion by FISH analysis

To validate the presence of *BCR-ABL1* fusion, we performed FISH analysis with dual color, single fusion probes on the patient’s bone marrow aspiration sample using the *BCR-ABL* FISH Probe kit (Jinpujia Medical, Beijing, China) according to the manufacturer’s instructions. DNA probes targeting the *BCR* (chromosome 22q11.2) and *ABL1* (chromosome 9q34) genes were labeled with green and red fluorescent dye, respectively. In normal cells, two green signals and two red signals were separated, representing that two probe-targeted sequences were located on different chromosomes. The presence of yellowish signal dots indicated the fusion events that resulted from the colocalization of *BCR*-targeting green fluorescent signals and *ABL1*-targeting red signals. The percent of cells with *BCR-ABL1* fusions was counted and the cutoff value for the *BCR-ABL1* fusion was set at 3% in our hospital.

### Detection of *BCR-ABL1* gene rearrangement by one-step RT-PCR

Routine fluorescence one-step RT-PCR was carried out to detect *BCR-ABL1* fusion transcripts. RNA from patient bone marrow aspiration samples was extracted using an RNeasy Kit (Qiagen, CA, USA), following the protocol provided by the manufacturer. RNA was purified by DNase I (Ambion, Applied Biosystems, TX, USA) digestion and was then subjected to one-step RT-PCR by a Leukemia Related Fusion Gene Detection Kit for *BCR-ABL* p210, p190, or p230 (Yuanqi Bio-Pharmaceutical, Shanghai, China). In each PCR process, a total volume of 25 μl reaction solution contains 3 μl template RNA, 2 μl multiplex Enzyme and 20 μl multiplex RT-PCR buffer. Amplification and detection were performed on a 7300 Real Time PCR System (ABI, USA). PCR procedure parameters were as follows: reverse transcription at 42 °C for 30 min, inactivation at 94 °C for 5 min, followed by 40 cycles of fluorescence detection at 94 °C for 15 s, and annealing at 60 °C for 60 s.

### Whole genome sequencing in a NGS platform

A genomic DNA (gDNA) library was constructed for sequencing following protocols of the TruSeq Nano DNA Library Preparation Kit (Illumina, San Diego, CA). Adaptors were ligated to library fragments sheared by Covaris (Covaris, Woburn, MA, USA) and were then subjected to PCR amplification. The quantitation and abundance determination of PCR amplicons were performed on Qubit 3.0 Fluorometer (Life Technologies, USA) and Agilent 2100 Bioanalyzer (Agilent Technologies, USA), respectively. WGS was performed on HiSeq X (Illumina, San Diego, CA), with the use of Illumina bcl2fastq software version 2.15 for base calling analysis.

## Abbreviations

AML: Acute myeloid leukemia
C/EBPα: CCAAT enhancer binding protein α
CML: Chronic myelogenous leukemia
CMML: Chronic myelomonocytic leukemia
CNL: Chronic neutrophilic leukemia
FISH: Fluorescence in situ hybridization
MDS: Myelodysplastic syndrome
MPNs: Myeloproliferative neoplasms
NGS: Next-generation sequencing
SH3: Src homology 3
TKI: Tyrosine kinase inhibitor
WBCs: White blood cells
WGS: Whole genome sequencing
